# Ang-(1-7) attenuates podocyte injury induced by high glucose *in vitro*

**DOI:** 10.20945/2359-3997000000643

**Published:** 2023-06-19

**Authors:** Jianxin Lu, Guixiang Chen, Guanghui Shen, Wenhao Ouyang

**Affiliations:** 1 Shanghai Jiao Tong University School of Medicine Shanghai Ninth People’s Hospital Division of Nephrology Shanghai P.R. China Division of Nephrology, Shanghai Ninth People’s Hospital Affiliated to Shanghai Jiao Tong University School of Medicine, Shanghai, P.R. China; 2 Children’s Hospital of Fudan University Paediatrics Research Institute Shanghai P.R. China Paediatrics Research Institute, Children’s Hospital of Fudan University, Shanghai, P.R. China; 3 Shanghai Xuhui Central Hospital Department of Clinical Laboratory Shanghai P.R. China Department of Clinical Laboratory, Shanghai Xuhui Central Hospital, Shanghai, P.R. China

**Keywords:** Renin angiotensin system (RAS), Ang-(1-7), Mas, podocyte, diabetic nephropathy

## Abstract

**Objective:**

The incidence of diabetic nephropathy (DN) is gradually increasing worldwide. Podocyte injury, such as podocyte apoptosis and loss of the slit diaphragm (SD)-specific markers are early pathogenic features of DN.

**Materials and methods:**

The cultured mouse podocytes were separated into a high glucose-treated (HG, 30mM) group to mimic DN *in vitro*, a low glucose-treated (LG, 5mM) group as a control and HG+ angiotensin-(1-7)(Ang-(1-7)) and HG+Ang-(1-7) + D-Ala7-Ang-(1-7) (A779, Ang-(1-7)/Mas receptor antagonist) experimental groups. The Cell Counting Kit-8 (CCK-8) method and flow cytometry was used to detect podocyte activity and podocyte apoptosis respectively. The expression of angiotensin type 1 receptor (AT1R), Mas receptor (MasR) and podocyte-specific markers were examined by q-PCR and Western blot, respectively.

**Results:**

The results showed that the decrease in podocyte activity; the increase in podocyte apoptosis; the decreased mRNA and protein expression of nephrin, podocin, WT-1 and MasR; and the upregulated expression of AT1R induced by HG could be reversed by Ang-(1-7). However, these effects were blocked by A779. The possible mechanisms of the Ang-(1-7)-mediated effect depended on MasR. In addition, the protective effect of Ang-(1-7) on podocyte activity was dose-dependent and most obvious at 10 µM. A779 had the greatest antagonistic action against Ang-(1-7) at a concentration of 10 μM.

**Conclusion:**

This study reveals that binding of Ang-(1-7) to its specific receptor MasR may counteract the effects of Ang II mediated by AT1R to significantly attenuate podocyte injury induced by high glucose. Ang-(1-7)/MasR targeting in podocytes may be a therapeutic approach to attenuate renal injury in DN.

## INTRODUCTION

Diabetic kidney disease (DKD), commonly termed diabetic nephropathy (DN), remains a predominant cause of end-stage renal disease (ESRD) in the Western world and places a large economic burden on society (1). Even in developing countries, the incidence of DN responsible for ESRD is gradually increasing.

Studies have shown a close relationship between reduced podocytes and foot process (FP) effacement and the progression of DN (2). Podocyte injury leads to the destruction of the integrity of the glomerular filtration barrier (GFB) and eventually to albuminuria in DN. SD is defined as a junction that bridging between long filtration slits, which is left between FPs of neighbouring podocytes interdigitate (3). Loss of expression of SD proteins, such as nephrin, podocin, and Wilms’ tumour protein-1 (WT-1), is thought to be one of the main pathogenic features of FPE in podocytes; accordingly, the restoration of SD proteins in podocytes is a potential treatment for DN (2,4).

The crucial roles of the intrarenal renin-angiotensin system (RAS) in the development and progression of DN have been recognized (5), and traditional RAS inhibitors (RASI), such as angiotensin-converting enzyme inhibitors (ACEIs)/angiotensin receptor blockers (ARBs), are currently considered to be effective treatments for DN. However, this therapeutics only partially delay the development and progression of DN (6); in addition, serious side effects are observed with dual RAS blockade (7). Inhibitors of sodium and glucose co-transporter 2 (iSGLT2), another new anti-diabetic drug, has been demonstrated to have cardiac and renal protective effects and has been used clinically (8). However, whether the combination of iSGLT2 and RASIs provides better cardio-renal clinical outcomes than iSGLT2 alone or not in patients with type 2 diabetes is unclear (9). Thus, it is critical to explore the specific mechanism of DN and develop promising novel agents targeting its pathogenesis to slow the progression of DN.

Recent advances regarding novel RAS-related pathways have indicated that these pathways are worthy of further study. Studies have shown that the pathogenesis of DN is related to the overactivation of angiotensin II (Ang II) (10). Ang-(1-7), a newly identified RAS component, binds to its specific receptor Mas (MasR) and antagonizes the effects of Ang II mediated by angiotensin type 1 receptor (AT1R) (11). The Ang-(1-7)/AT_7_-MasR antagonist D-Ala7-Ang-(1-7) (DAL or A779) abolishes the inhibitory actions of Ang-(1-7). Research has been executed to explore the renoprotective effects of the activation of the Ang-(1-7)/Mas pathway using MasR agonists in experimental models of diabetes (12). However, targeting the kidneys is more important than targeting cyclic RAS to conquer DN (13). The direct effect of Ang-(1-7) on podocytes in intrarenal local RAS in evaluating possible mechanisms involved in kidney disease has not been studied. Therefore, this topic was the focus of the current investigation.

## MATERIALS AND METHODS

### Cell culture

Conditionally immortalized mouse podocytes (14) were cultured as previously described (15). The culture flasks were precoated with type I collagen (Sigma-Aldrich) at 37 °C for 1 h. After the podocytes were routinely resuscitated, the cells were inoculated and cultured at 33 °C in complete RPMI-1640 medium (foetal bovine serum [FBS], Gibco BRL, Gaithersburg, MD, USA) supplemented with 10% FBS (Gibco BRL), 100 U/mL penicillin (Gibco BRL), 100 U/mL streptomycin (Gibco BRL), and 50 U/mL recombinant interferon-γ (IFN-γ, Sigma-Aldrich) in a 5% CO_2_ saturated humidified incubator to promote proliferation. The culture medium was changed every 2 to 3 days, and IFN-γ was reduced to 20 U/mL. Routine transfer culture was performed until IFN-γ was maintained at 10 U/mL. Subsequently, the proliferated podocytes were transferred to 37 °C and deprived of IFN-γ for 10 to 14 days to induce differentiation until maturation. The podocyte shape/structure was observed under an inverted phase-contrast microscope, and differentiated matured podocytes at 37 °C were used for follow-up experiments.

### Cell treatments

Matured mouse podocytes were inoculated into 96-well plates at a density of 30%. The medium was then replaced with complete RPMI-1640 medium containing 10% FBS and lacking IFN-γ and the cells were incubated at 37 °C for 5 days. Approximately 10^6^ cells were synchronized into quiescence by maintenance in serum-free RPMI-1640 medium for 24 h and then cotreated with one or combinations of the following: low glucose (5 mM, LG), high glucose (30 mM, HG), and Ang-(1-7) (0/0.01/0.1/1/10 μM, Sigma-Aldrich) for 24 h. The optimal concentration of Ang-(1-7) was selected for subsequent experiments through preliminary experiments, and then cells were pretreated with or without different concentrations of A779 (1 μM/10 μM/100 μM, Sigma-Aldrich) for 15 minutes to select the best concentration of A779 for subsequent experiments. Each group podocytes were provided with three duplicates and each reaction was repeated in triplicate.

### Cell survival (cell counting Kit-8, CCK-8) assay

To determine the effects of different treatments on podocyte activity, the viability of cultured mouse podocytes was measured using a CCK-8 assay (BBI Life Sciences, Shanghai, China). The culture medium of the treated podocytes in 96-well plates was replaced with 10 μL of CCK-8 solution, and the cells were incubated for 1 hour away from light in a 37 °C incubator with 5% CO_2_. The absorbance values were then measured at a wavelength of 450 nm using a microplate reader (Biotek EPOCH2, USA). The absorbance of the blank wells with only growth medium was subtracted from the values for the wells with cells. Each group of podocytes was tested in triplicate, and the experiments were all repeated three times.

### Flow cytometry analysis

After treatment, the podocytes were collected for apoptosis detection. Cells were digested with trypsin to generate a single-cell suspension and centrifuged at 1500 rpm/min for 5 min. The supernatant was removed, and the cells were collected. Then, the cells were washed with phosphate-buffered saline (PBS) solution twice and centrifuged at 1500 rpm/min for 5 min again. After the final rinse step, the cells were resuspended in 100 μL of binding buffer (1x) and then incubated with 5 μL of Annexin V-PE and 10 μL of 7-AAD reagent at room temperature in the dark for 30 min. The mixture was diluted with 385 μL of binding buffer (1x) and analysed using flow cytometry within 2 hours. These assays were performed with an Annexin V-PE/7-AAD Apoptosis Detection Kit (YEASEN, Shanghai, China) following the manufacturer’s instructions.

### Quantitative real-time PCR (qRT-PCR)

qRT-PCR was used to evaluate the mRNA alterations of the podocyte SD proteins nephrin, podocin, and WT-1 as well as those of AT1R and MasR. Total RNA was extracted from treated podocytes with TRIzol Reagent (15596-018; Invitrogen) and then quantified on a spectrophotometer (Shunyu Hengping, Shanghai, China, Model 752). The extracted RNA was reverse-transcribed into cDNA by using a first-strand cDNA synthesis kit (EP0733; Thermo Scientific™, USA) according to the manufacturer’s instructions, and the cDNA was stored at -20 °C until use. qRT-PCR was performed in a PCR system (ABI StepOnePlus) with a 20-µL reaction volume containing 2 µL of cDNA, 1 µL of Ex Taq™ (DRR100A, Takara), primers for nephrin, podocin, WT-1, AT1R, MasR or *GAPDH*, and 10 µL of SG Fast qPCR Master Mix (B639273, Sangon Biotech, Shanghai, China). The primer sequences were as follows: nephrin, *5*′*-GATGCGGAGTACGAGTGCC-3*′ and *5*′*-GGGGAACTAGGACGGAGAGG-3*′; podocin, *5*′*-GACCAGAGGAAGGCATCAAGC-3*′ and *5*′*-GCACAACCTTTATGCAGAACCAG-3*′; WT-1, *5*′*-GAGAGCCAGCCTACCATCC-3*′ and *5*′*-GGGTCCTCGTGTTTGAAGGAA-3*′; AT1R, *5*′*-ATGCTTGGGGCAACTTCACTA-3*′ and *5*′*-GCAGCAAGAGAAGGGCTTCA-3*′; and MasR, *5*′*-AGAAATCCCTTCACGGTCTACA-3*′ and *5*′*-GTCACCGATAATGTCACGATTGT-3*′. The thermocycling program included 41 cycles: initial denaturation at 95 °C for 3 min followed by 40 cycles of melting at 95 °C (7 sec), annealing at 57 °C (10 sec) and extension at 72 °C (15 sec). The relative mRNA expression levels of the target genes were normalized to the glyceraldehyde 3-phosphate dehydrogenase (GAPDH) levels. The specificity of the amplification product was verified by melting curve analysis. Experiments were performed in triplicate for each gene and were repeated three times using independent biological replicates.

### Western blot analysis

Western blotting (WB) was performed to assess the protein expression of the podocyte SD proteins nephrin, podocin, and WT-1 as well as the proteins AT1R and MasR, as previously described (16). Briefly, the podocytes were washed twice with precooled PBS and collected in RIPA lysis buffer (P0013B, Beyotime, Shanghai, China) containing a protease inhibitor cocktail (ST506, Sigma, USA) and phosphatase inhibitors (S1873, Beyotime, Shanghai, China) at 4 °C for 30 min with oscillation every 10 min. Each decomposed sample was ultrasound-treated with an ultrasonic homogenizer at 25% power 12 times for one second at one-second intervals. Then, the cell debris was centrifuged for 10 minutes at 13000 rpm and 4 °C. The protein concentration of the supernatant was quantified using a BCA Protein Assay Kit (P0010, Beyotime, Shanghai, China). After boiling at 100 °C for 10 min in 3× loading buffer, protein samples (40 µg) from each group were loaded and separated by 10% sodium dodecyl sulfate-polyacrylamide gel electrophoresis and transferred to polyvinylidene difluoride membranes (Millipore). The membranes were blocked at room temperature with 5% nonfat dry milk in Tris-buffered saline with Tween-20 (TBS-T) for 30 min and then incubated with the following primary antibodies at appropriate dilutions at 4 °C overnight: anti-nephrin (1:500, Santa Cruz, USA), anti-podocin (1:1000, Abcam, USA), anti-WT-1 (1:500, Santa Cruz, USA), anti-AT1R (1:250, Santa Cruz, USA), anti-MAS1 (1:500, Santa Cruz, USA) and anti-GAPDH (1:20000, Sigma-Aldrich, USA). After washing 6 times with TBS-T for 5 min each time, the blots were incubated with their corresponding horseradish peroxidase-linked secondary antibodies (1:5000, Boster, Wuhan, China) for 2 hours at 37 °C. The immunoreactive bands were visualized by enhanced chemiluminescence (ECL) methods (NCI5079, Thermo Fisher Scientific, Waltham, MA, USA). The images were developed on Kodak Omat X-ray films and normalized to tubulin with computerized analysis.

### Statistical analysis

Each group podocytes were provided with three duplicates and all experiments were repeated at least three times (n = 9). The final data are expressed as the means ± standard deviations and were analysed using GraphPad Prism 7.0 (GraphPad Software Inc., San Diego, CA, USA). Statistical analyses were performed by one-way analysis of variance (ANOVA) or Student-Newman-Keuls (SNK-q) test. All *P* values were two-tailed, and *P* < 0.05 was considered to indicate a statistically significant difference between the compared groups.

## RESULTS

### Effects of different concentrations of intervention reagents on podocyte activity detected by the CCK-8 method

**Figure 1:**
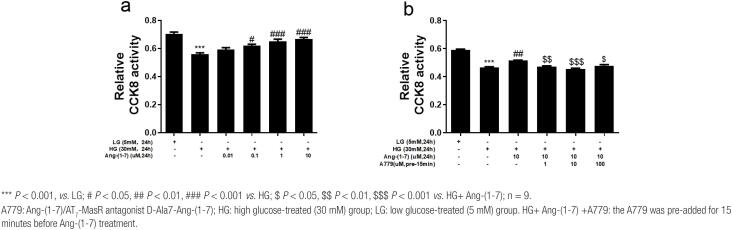
A CCK-8 assay was performed to detect the effects of different concentrations of Ang-(1-7) (a) and A779 (b) on the activity of podocytes treated with HG.

The CCK-8 assay results showed that the podocyte activity was significantly suppressed by HG (0.558 ± 0.018, *P* < 0.001) compared with LG (0.71 ± 0.15); addition of Ang-(1-7) at different concentrations (0.01/0.1/1/10 μM) (0.593 ± 0.022, *P* > 0.05/0.621 ± 0.016, *P* < 0.05/0.650 ± 0.029, *P* < 0.001/0.666 ± 0.021, *P* < 0.001) gradually attenuated the HG-mediated suppression of podocyte activity (0.558 ± 0.018). In general, the decrease in podocyte activity induced by HG was reversed by Ang-(1-7) in a concentration-dependent manner. Furthermore, Ang-(1-7) had the greatest protective effect on podocyte injury induced by HG at a concentration of 10 µM (Figure 1a).

Subsequently, HG-treated podocytes were pretreated with different concentrations of A779 (1 μM/10 μM/100 μM) for 15 minutes and then cocultured with 10 µM Ang-(1-7). The podocyte activity in the HG+ Ang-(1-7) + A779 group (0.472 ± 0.008, *P* < 0.01/0.454 ± 0.011, *P* < 0.001/0.478 ± 0.014, *P* < 0.05) was suppressed compared with that in the Ang-(1-7) +HG intervention group (0.516 ± 0.004). This suggested that A779 antagonized the protective effect of Ang-(1-7) on podocytes, thus aggravating podocyte injury induced by HG. Moreover, A779 had the greatest antagonistic action against Ang-(1-7) at a concentration of 10 μM (Figure 1b).

### Protective effect of Ang-(1-7) against podocyte apoptosis induced by HG

**Figure 2:**
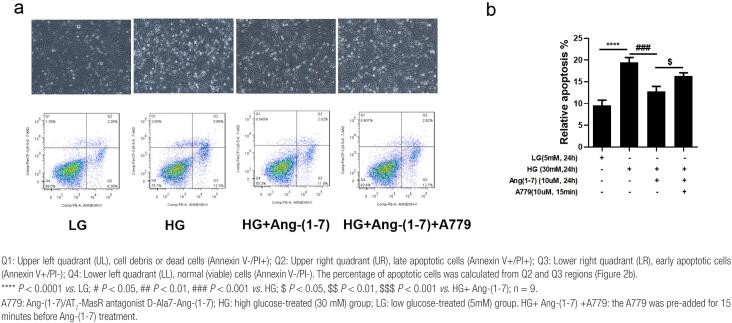
Flow cytometry was used to detect podocyte apoptosis (a) and the differences in relative apoptotic rates (b) among the LG, HG, HG+ Ang-(1-7) and HG+ Ang-(1-7) +A779 groups.

In the flow cytometry assay, we found that HG exposure markedly triggered apoptosis of podocytes (*P* < 0.0001) compared with that in the LG group; however, 10 µM Ang-(1-7) significantly decreased the podocyte apoptosis induced by HG (*P* < 0.001 *vs.* HG), while A779 reversed this effect and led to an apparent increase in the apoptosis rate (*P* < 0.05 *vs.* HG+ Ang-[1-7] group) (Figure 2a–b). Pretreatment with 10 μM A779 thus attenuated the protective effect of Ang-(1-7) against HG-triggered podocyte apoptosis. This result was consistent with the experimental results obtained with the CCK-8 method.

### Effect of Ang-(1-7) on the mRNA expression of the SD proteins nephrin, podocin, and WT-1 as well as AT1R and MasR in HG-treated podocytes

**Figure 3:**
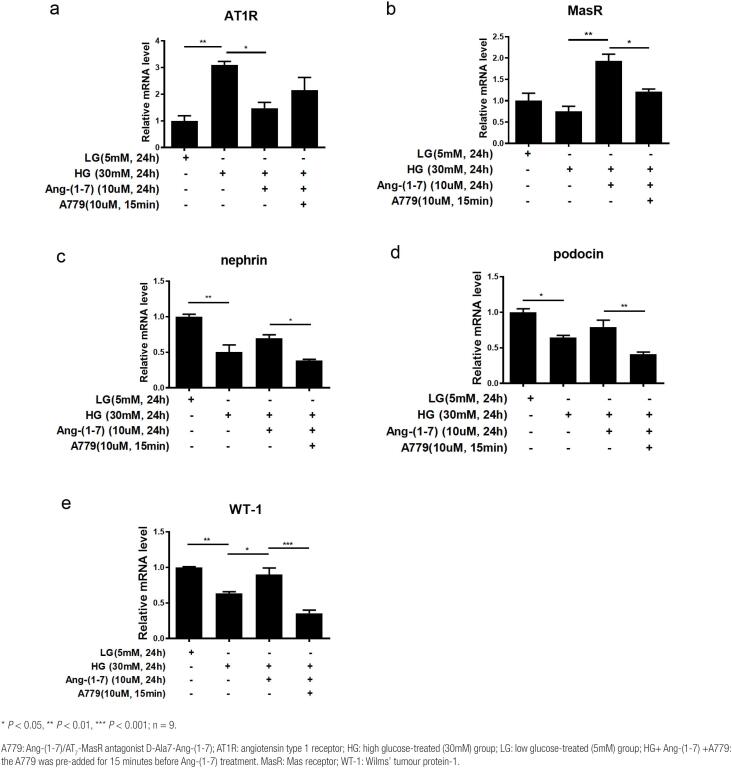
q-PCR detection of the effects of Ang-(1-7) on the mRNA expression of AT1R (a), MasR (b), and the podocyte SD proteins nephrin (c), podocin (d), and WT-1 (e) in HG-treated podocytes.

To investigate the protective roles of Ang-(1-7) and its possible targets of action in DN, alterations in transcript levels were further studied. The results of q-PCR detection showed that the mRNA expression of AT1R in podocytes was significantly higher in the HG group (*P* < 0.01) than in the LG control group. However, the effect was strikingly abolished after addition of 10 µM Ang-(1-7); AT1R mRNA expression was notably repressed (*P* < 0. 05 *vs.* HG). Nevertheless, pretreatment with 10 μM A779 to antagonize the effect of Ang-(1-7) reversed the increase in AT1R mRNA expression in podocytes, although the difference was not significant (*P* > 0.05 *vs.* HG+ Ang-[1-7] group) (Figure 3a).

The mRNA expression of MasR in podocytes was slightly suppressed in the HG-stimulated group compared with the LG group (*P* > 0.05). The addition of Ang-(1-7) led to an evident increase in MasR mRNA expression (*P* < 0.01 *vs.* HG group), but this effect was reversed by A779; the mRNA expression of MasR was evidently decreased (*P* < 0.05 *vs.* HG+ Ang-[1-7] group) (Figure 3b).

As illustrated in Figure 3c–e, the mRNA expression of the podocyte markers nephrin, podocin and WT1 showed the same trend as that of MasR. The mRNA levels of nephrin, podocin and WT1 were significantly lower in HG-treated podocytes than in LG-treated podocytes (nephrin, WT-1: *P* < 0.01; podocin: *P* < 0.05). Intervention with Ang-(1-7) promoted an increase in podocyte SD marker expression to some extent (nephrin, podocin: *P* > 0.05; WT-1: *P* < 0.05 *vs.* HG), but the protective effect was abrogated by inhibiting Ang-(1-7) with A779, which resulted in a striking decrease in podocyte marker mRNA expression (nephrin: *P* < 0.05, podocin: *P* < 0.01, WT-1: *P* < 0.001 *vs.* HG+ Ang-[1-7]).

### Effect of Ang-(1-7) on the protein expression of the SD proteins nephrin, podocin, and WT-1 as well as AT1R and MasR in HG-treated podocytes

**Figure 4:**
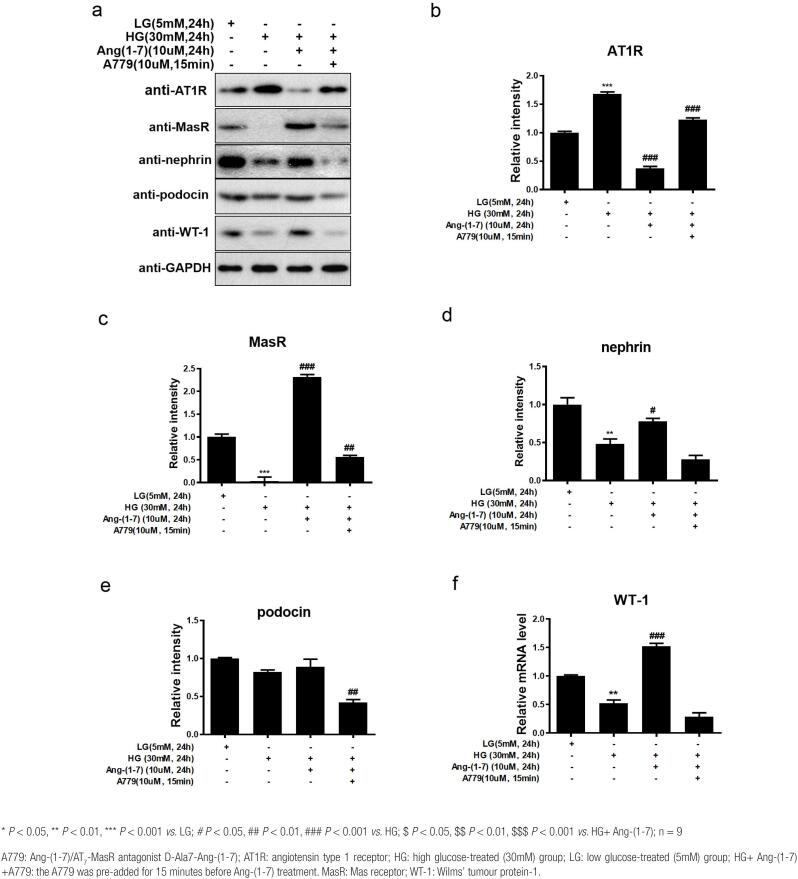
Western blot analysis (a) of the effects of Ang-(1-7) on the protein expression of AT1R (b) and MasR (c) and the SD proteins nephrin (d), podocin (e), and WT-1 (F) in HG-treated podocytes.

To further explore the mechanism by which Ang-(1-7) protected against HG-induced podocyte injury, the protein expression of AT1R, MasR, and the podocyte SD proteins nephrin, podocin, and WT-1 was examined by Western blot analysis (Figure 4a). The results revealed that the change trends were basically consistent with the mRNA expression results. Compared to the LG group, the HG group of podocytes exhibited significantly higher AT1R protein expression (*P* < 0.01). AT1R protein expression was evidently decreased in the HG+ Ang-(1-7) group (*P* < 0.01 *vs.* HG), but it was markedly enhanced again in the HG+ Ang-(1-7) + A779 group (*P* < 0.01 *vs.* HG+ Ang-[1-7] and HG) (Figure 4b).

MasR protein expression in HG-treated podocytes was remarkably lower than that in the LG-treated podocytes (*P* < 0.01). However, Ang-(1-7) had the opposite effect: in the HG+ Ang-(1-7) group, the protein expression of MasR was overtly increased (*P* < 0.01 *vs.* HG). A779 antagonized the effect of Ang-(1-7), prominently decreasing MasR protein expression in the HG+ Ang-(1-7) + A779 group (*P* < 0.01 *vs.* HG+ Ang-[1-7]; *P* < 0.05 *vs.* HG) (Figure 4c).

Similarly, the protein expression levels of the podocyte markers nephrin and WT-1 in HG-stimulated podocytes were also remarkably lower than those in LG-stimulated podocytes (*P* < 0.05); nevertheless, they were all apparently promoted in the HG+ Ang-(1-7) group (nephrin: *P* < 0.05 *vs.* HG; WT-1: *P* < 0.01 *vs.* HG) (Figure 4d–f). Podocin protein expression displayed the same tendency among the LG, HG and HG+ Ang-(1-7) groups, although there were no significant differences (Figure 4e). In addition, the protein expression of these podocyte markers in the HG+ Ang-(1-7) + A779 group showed a pronounced decrease (nephrin: *P* < 0.05 *vs.* HG+ Ang-[1-7]; WT-1: *P* < 0.01 *vs.* HG+ Ang-[1-7]; podocin: *P* < 0.05 *vs.* HG and HG+ Ang-[1-7]) (Figure 4d–f). All these data demonstrated that treatment with Ang-(1-7) downregulated AT1R expression while upregulating the expression of MasR and the SD proteins nephrin, podocin, and WT-1 in mouse podocytes treated with HG. The Ang-(1-7)/AT_7_-MasR antagonist A779 reversed these effects.

## DISCUSSION

Podocyte injury and/or SD disruption, detachment, reduced podocyte density and number, and podocyte apoptosis, may play an important role in the pathogenesis of DN (17-19). However, the potential mechanism of podocyte injury in DN has not been fully clarified. The podocyte-specific markers nephrin, podocin and WT-1, components of the SD, maintain the normal structure and function of podocytes (20). In this regard, therapeutic strategies to target podocyte injury hold considerable promise for the treatment of DN. In the present study, we established a podocyte injury model induced by HG *in vitro* to mimic the HG environment of DN.

The RAS is a counterregulatory system that consists of two axes. It is divided into the classical axis composed of angiotensin-converting enzyme (ACE)/Ang II/AT1R and the novel reverse regulatory axis comprising angiotensin-converting enzyme 2 (ACE2)/Ang-(1-7)/MasR (21). AT1R is expressed in a variety of kidney cells, including podocytes, and the activated ACE/Ang II/AT1R axis can induce vasoconstriction and multiple signal transduction pathways that lead to podocyte damage and renal disease. An antagonist of AT1R can prevent streptozotocin-induced DN (22). In addition to the systemic circulating RAS, persistent intrarenal activation of Ang II is associated with arterial hypertension and chronic kidney disease (CKD) (21). Ang II is also involved in the downregulation of SD proteins in diabetes, but the exact mechanism is still unclear (4).

Currently, Ang-(1-7), a neoteric component of the RAS acts on the specific receptor MasR to exert an effect opposing that of Ang II-AT1R binding. Accumulating evidence has demonstrated that Ang-(1-7) relieves the progression of DN in animal models (23,24). In one study, circulatory Ang-(1-7) had the same anti-proteinuric effect as the ACEI lisinopril, which was used for comparison, and alleviated glomerular fibrosis and inflammation even better than lisinopril (25). Circulatory Ang-(1-7) also ameliorates impairment in podocyte expression of nephrin, which maintains SD integrity and the glomerular filtration rate, and nestin, a protein implicated in the organization of the cytoskeleton, and limits podocyte loss. These beneficial effects are all similar to those of ACEIs (26). When circulatory Ang-(1-7) is combined with lisinopril, the anti-proteinuric effect is better than that of the ACEI alone, exhibiting additive renoprotective action as well as better protection against changes in podocyte markers and GFB (27). Despite their importance, the exact roles of Ang-(1-7) and MasR, especially in the intrarenal RAS targeted to podocytes in DN, is not clearly understood. This topic strongly stimulated our interest because the evidence suggests that Ang-(1-7) or other Mas agonists might be useful in the development of therapeutic agents to counteract the adverse role of Ang II/AT1R in DN. The present results strongly support it. To further validate the protective effect of Ang-(1-7) on podocytes, A779, a specific antagonist of Ang-(1-7)/MasR, was used in the following experiments. These results indicated that A779 intervention impeded the favourable effect of Ang-(1-7) on podocyte activity. Of note, the antagonistic action of A779 against Ang-(1-7) also manifested dose-dependently, and 10 μM A779 presented the greatest antagonism against Ang-(1-7).

In response to hyperglycaemic conditions, renal cells begin to secrete Ang II, which results in intrarenal Ang II being greatly elevated compared with circulating Ang II in DN. This redistribution of Ang II not only has a considerable impact on ion transport in the kidneys but also has a causal influence on various typical characteristics of DN, including podocyte injury and apoptosis (28,29). Activation of the ACE2/Ang-(1-7)/MasR axis plays a protective role by reducing oxidative stress, inflammation, cell proliferation, and fibrosis (30), which contributes to podocyte apoptosis.

Nephrin is thought to be the backbone of the SD (31) and the downregulation of nephrin is an early feature of human DN (4). Decreased podocin activity or expression also mediates podocyte injury (32,33). As two key functional molecules in podocyte SD, these proteins can interact with each other to affect the stability and integrity of SD. Therefore, the abnormal expression and distribution of nephrin and podocin could be a mechanism and a therapeutic target for proteinuria in DN. WT-1 is only expressed in podocytes of mature kidneys (34). Decreased WT-1 in podocytes in DN, which represents podocyte detachment and loss, occurs in patients with either type 1 or type II diabetes (26,35). Our findings suggested that A779 intervention impeded the effect of Ang-(1-7) on the expression of these factors and confirmed that this effect depended on MasR. These effects also prove that Ang-(1-7) acts as a counterregulator of the AT1R-mediated effects of Ang II. AT1R is believed to mediate most functions of Ang II in the classical RAS (36), and AT1R activation is known to downregulate nephrin expression (37).

Certainly, the limitation of the study should be fully considered. Neither rodent models nor cell models in vitro are ideal for transposition data to humans. Due to the complexity of the pathogenesis of diabetes, the podocyte injury model induced by high glucose in this study cannot fully represent the actual internal environment of diabetes, in which many other factors may be acting. The effects of other factors on podocytes and even kidneys in diabetes and the interactions between these factors need to be further studied in vitro, in vivo experiments in animals and clinical trials in humans. However, these present data provided preliminary evidence that the Ang-(1-7)/MasR axis plays an important role in regulating podocyte survival and SD integrity upon HG-induced podocyte injury. The underlying mechanisms involving the interactions of receptors in the RAS and related signal transduction pathways will be taken into more detailed consideration in future studies.

Despite the proven efficacy of routine clinical strategies, such as ACEI, ARB, iSGLT2 treatment, and glycaemic and blood pressure control, for reducing proteinuria and slowing the progression of DN, these treatments are still not enough to prevent kidney damage (38). Therefore, podocytes may be a therapeutic target for repair and recovery. The above research results provide a valuable experimental basis for the clinical application of Ang-(1-7) in the prevention and treatment of DN. Hence, supplementation with Ang-(1-7) with or without either an AT1R antagonist or an ACEI may be a more effective therapeutic approach.

In conclusion, Ang-(1-7) significantly increased podocyte activity in a dose-dependent manner, reduced podocyte apoptosis, and upregulated and restored the expression of podocyte SD proteins. The possible mechanisms of the Ang-(1-7)-mediated effect depended on Mas receptor, which can be confirmed in subsequent studies using A779 as an Ang-(1-7)/Mas receptor antagonist. Ang-(1-7)/MasR may play an antagonistic role against Ang-/AT1R and has potential therapeutic value for DN. However, the current study was just a preliminary study; the underlying mechanisms involving the interactions of receptors in the RAS and related signal transduction pathways will be taken into more detailed consideration in future studies.
